# Changes in the U.S. Primary Standards for the Air Kerma From Gamma-Ray Beams

**DOI:** 10.6028/jres.108.031

**Published:** 2003-10-01

**Authors:** Stephen M. Seltzer, Paul M. Bergstrom

**Affiliations:** National Institute of Standards and Technology, Gaithersburg, MD 20899

**Keywords:** air kerma, cavity chamber, electron stopping-power ratio, exposure, humidity correction, Monte Carlo, national standard, photon energy-absorption coefficient ratio, radiative-loss correction, wall correction

## Abstract

Monte Carlo photon-electron transport calculations have been done to derive new wall corrections for the six NBS-NIST standard graphite-wall, air-ionization cavity chambers that serve as the U.S. national primary standard for air kerma (and exposure) for gamma rays from ^60^Co, ^137^Cs, and ^192^Ir sources. The data developed for and from these calculations have also been used to refine a number of other factors affecting the standards. The largest changes are due to the new wall corrections, and the total changes are +0.87 % to +1.11 % (depending on the chamber) for ^60^Co beams, +0.64 % to +1.07 % (depending on the chamber) for ^137^Cs beams, and −0.06 % for the single chamber used in the measurement of the standardized ^192^Ir source. The primary standards for air kerma will be adjusted in the near future to reflect the changes in factors described in this work.

## 1. Introduction

The National Institute of Standards and Technology (NIST), formerly the National Bureau of Standards (NBS), maintains the primary standards for exposure and air kerma for x rays and gamma rays. As is the case for other National Metrology Institutes (NMIs), our primary standards for ^60^Co and ^137^Cs gamma-ray fields and for the gamma rays from a number of ^60^Co, ^137^Cs, and ^192^Ir brachytherapy sources are derived from measurements using graphite-wall, air-ionization, cavity chambers, based on Bragg-Gray theory. The final value of the air kerma (or exposure) depends on the values assigned to a number of factors involved in the conversion of the measured results to air kerma (or exposure).

Kerma, *K*, is defined [1] as the quotient of d*E*_tr_ by dm, where d*E*_tr_ is the sum of the initial kinetic energies of all the charged particles liberated by uncharged particles (in our case, photons) in a mass d*m* of material. Thus,

$$K=\frac{dE_{\text{tr}}}{dm}.$$ (1)

The exposure, *X*, is defined [1] as the quotient of d*Q* by dm, where d*Q* is the absolute value of the total charge of the ions of one sign produced in air when all the electrons and positrons liberated or created by photons in air of mass d*m* are completely stopped in air. Thus,

$$X=\frac{dQ}{dm}.$$ (2)

The SI unit of exposure is C kg^−1^; however, the older unit of Roentgen (R) is still used by some, where 1 R = 2.58 × 10^−4^ C kg^−1^. The quantities exposure and air kerma can be related through use of the mean energy per unit charge, *W/e*, where *W* is the mean energy expended in air per ion pair formed when the initial kinetic energy of a charged particle is completely dissipated in the air, and *e* is the elemental charge. Then

$$K_{\text{air}}=X\cdot (W/e)/(1-\bar{g}).$$ (3)

The quantity *g* is the fraction of the kinetic energy of electrons (and positrons) liberated by the photons that is lost in radiative processes (mainly bremsstrahlung) in air. In Eq. (3), $\bar{g}$ is the mean value of *g* averaged over the distribution of the air kerma with respect to the electron energy. The values for $\bar{g}$ adopted by NBS-NIST for the conversion to air kerma have been 0.0032 for ^60^Co, 0.0016 for ^137^Cs and 0.0000 (by omission) for ^192^Ir. The value of *W/e* for dry air currently adopted by the international measurement system is 33.97±0.05 J/C [2].

Bragg-Gray cavity theory [3] relates the ionization per unit mass in a small gas cavity to the energy absorbed per unit mass in the surrounding medium:

$$D_{m}=J_{g}W_{g}\frac{\bar{{\left(S/\rho \right)}_{m}}}{\bar{{\left(S/\rho \right)}_{g}}},$$ (4)

where *D*_m_ is the absorbed dose in the medium surrounding the cavity, *J*_g_ is the ionization per unit mass in the cavity, *W*_g_ is mean energy expended in the gas to produce an ion pair, and $\frac{\bar{{\left(S/\rho \right)}_{m}}}{\bar{{\left(S/\rho \right)}_{g}}}$ is the ratio of the mean electron-fluence-weighted electron mass stopping power of the medium to that of the gas. This relation is valid provided that the medium (or wall) is thick enough to exclude secondary electrons generated in material other than the medium (wall) from entering the cavity, and that the cavity is small enough so as not to perturb the secondary electron fluence.

The absorbed dose in the gas, in the absence of the medium (wall) is

$$D_{g}=D_{m}\frac{\bar{{\left(\mu_{\text{en}}/\rho \right)}_{g}}}{\bar{{\left(\mu_{\text{en}}/\rho \right)}_{m}}},$$ (5)

where $\frac{\bar{{\left(\mu_{\text{en}}/\rho \right)}_{g}}}{\bar{{\left(\mu_{\text{en}}/\rho \right)}_{m}}}$ is the ratio of the mean photon-energy-fluence-weighted photon mass energy-absorption coefficient of the gas to that of the medium. Combining Eqs. (2) to (5), one obtains the absorbed dose (and closely related quantities) in the gas from ionization measurements with a cavity chamber under conditions that now assure the requisite charged-particle equilibrium. For graphite as the wall material and air as the cavity gas, one can then write for the air kerma *K*_air_:

$$K_{\text{air}}=\frac{Q_{\text{air}}}{V\rho_{\text{air}}}\frac{\left(W_{\text{air}}/e\right)}{1-\bar{g}}\frac{{\bar{\left(S/\rho \right)}}_{\text{graphite}}}{{\bar{\left(S/\rho \right)}}_{\text{air}}}\frac{{\bar{\left(\mu_{\text{en}}/\rho \right)}}_{\text{air}}}{{\bar{\left(\mu_{\text{en}}/\rho \right)}}_{\text{graphite}}}\prod_{i}k_{i},$$ (6)

where *Q*_air_ is the measured ionization charge, *V* is the cavity volume, *ρ*_air_ is density of the (dry) air in the cavity, and *k_i_* are the correction factors required to correct the measured charge for experimental perturbations. Note that for later convenience, we adopt for the ratio of spectrum-weighted averages the shorthand notation ${\left(\bar{x}\right)}_{b}^{a}\equiv \frac{{\left(\bar{x}\right)}_{a}}{{\left(\bar{x}\right)}_{b}}$. The correction factors in Eq. (6) include *k*_sat_ for the loss of collected ionization due to recombination, *k*_stem_ for the effects of chamber-stem scatter, *k*_h_ for the effects of water vapor in the air (humidity), and *k*_wall_ for the effects of photon attenuation and scatter in the chamber wall.

The goal of such measurements is to directly realize the air kerma (or exposure) at a point in the gamma-ray field. The chamber (mainly its walls) perturbs such a measurement. The wall correction is intended to account for the effects of attenuation of the incident primary photons in the chamber wall (and cavity air) and to remove the contribution to the recorded ionization from any photon interaction other than the first interaction in the chamber wall (or cavity air). Thus the application of *k*_wall_ renders the measurement as that corresponding to a point in air in the absence of the chamber. The empirical method to estimate *k*_wall_ has been to measure the ionization charge (or current) as a function of wall thickness for a fixed cavity size (but for wall thicknesses no smaller than the minimum required to exclude secondary electrons generated from outside the wall). The results are then linearly extrapolated to zero wall thickness, obtaining *k*_extrap_, under the assumption that attenuation and scattering are thus eliminated. A further correction, k_CEP_, is applied to account for the depth in the wall at which the electrons entering the cavity are produced. The final *experimental* wall correction is then $k_{\text{wall}}^{\exp}=k_{\text{extrap}}k_{\text{CEP}}$.

For more than a decade, work at National Research Council (NRC), Canada [4–8] has suggested that the use of $k_{\text{wall}}^{\exp}$ based on linear extrapolation is incorrect, and proposed instead the use of results from Monte Carlo photon-electron transport calculations. At the 14th meeting in May 1999 of the Consultative Committee on Ionizing Radiation, Section I [CCRI(I)], of the International Committee on Weights and Measures, a working group was established to study the implications of using *k*_wall_ correction factors from Monte Carlo calculations. The members of the working group included representatives from NIST and a number of other NMIs. Of primary concern are the possible effects on air-kerma standards for ^60^Co gamma rays that have served as the basis for calibrations of instruments used in radiation-therapy beams. Preliminary results developed at NIST for ^60^Co gamma-ray beams were reported to the 15th meeting of the CCRI(I) in May 2001. The present report gives the final results, intended as the basis for the formal revision of NIST gamma-ray air-kerma standards. The implementation of the changes is scheduled for the near future, upon formal notification of all concerned parties.

**Fig. 1:**
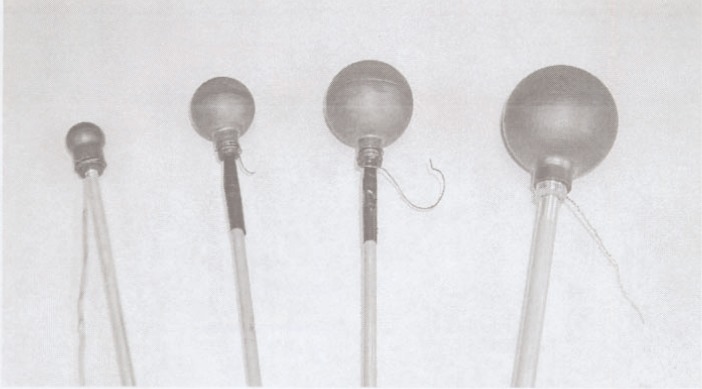
NBS-NIST standard graphite-walled, air-ionization cavity chambers.

Our primary-standard measurements are made using a suite of spherical, graphite-wall, air-filled, cavity chambers. Representative chambers are shown in Fig. 1. The chambers, their use, and their results have been rather completely described by Loftus [9, 10], Loftus and Weaver [11], and Weaver, Loftus and Loevinger [12]. Earlier (and essentially unpublished) modifications to the factors used by NBS in exposure standards, based on recommendations of the 11th meeting in April 1985 of the Consultative Committee on Ionizing Radiation, Section I [CCEMRI(I)], of the International Committee on Weights and Measures, were made effective on 1 January 1986. Those modifications, made in light of then-newer information on photon mass energy-absorption coefficients [13], on electron mass electronic (collision) stopping powers [14, 15], and on humidity corrections to air-ionization-chamber results [16], are given in Table 1.

The modified Loftus-Weaver correction factors are summarized in Table 2. The chamber designations indicate the nominal cavity volume in cm^3^ of the chamber; the three 50 cm^3^ chambers have different wall thicknesses. Note that in Table 2 the factor $k_{\text{wall}}^{\exp}$ is the the product of the “extrapolated” wall-attenuation factor and the correction for the “center of electron production” (*k*_CEP_ = 0.9950), as given by Loftus and Weaver [10]. Although the 1986 NBS adjustment factors were given only to three significant figures, the use of four significant figures by Loftus and Weaver has been retained for the modified stopping-power and the energy-absorption ratios given in Table 2.

In what follows, Monte Carlo calculations for the NBS-NIST graphite-wall, air-ionization, cavity chambers and the analyses of results are described. Although *k*_wall_ is the main subject of this work, the information used in that determination provides the opportunity to re-evaluate (and largely confirm) also the adopted values of $\bar{g}$, (μ¯enρ)graphiteair, (S¯ρ)airgraphite, and *k_h_*.

## 2. Monte Carlo Calculations

NRC’s work has been based on use of the EGS4 electron-photon Monte Carlo transport code, and other national metrology institutes have indicated the use of this code as well as MCNP, and PENELOPE. As NIST developed the ETRAN Monte Carlo code, which provides the physics engine for the Integrated Tiger Series (ITS) codes and, in turn, the electron-transport algorithms for MCNP4, it was decided to use the ACCEPT module from ITS version 3.0 [17] for the bulk of the calculations. This choice was made both because the first author of this report understands the ITS code better and because it might provide independent results for comparison to those from EGS and other Monte Carlo codes. In addition to a few minor updates to ITS3, the ACCEPT code was modified to include correlated scoring of the energy deposited by (a) all secondary electrons and their progeny from primary and scattered photons, i.e., the usual total energy deposition, denoted here as 〈*ε*〉 = 〈*ε*_0_ + *ε*_s_〉, where *ε*_s_ is the energy deposition from all secondary electrons and their progeny produced by photons scattered in the chamber; (b) all secondary electrons and their progeny from only the primary photons, i.e., “first-collision” energy deposition, denoted here as 〈*ε*_0_〉; and (c) the first-collision energy deposition, corrected for attenuation of the primary photon, i.e., “unattenuated first-collision” energy deposition, denoted here as 〈*e*^+^*^µt^ε*_0_〉. All scores (a, b, and c) are done simultaneously in each history, so the results are completely correlated, which greatly reduces the statistical uncertainty in the various ratios. It is instructive to separate the theoretical wall correction into two factors:

$$\begin{array}{l} k_{\text{at}}=\langle e^{+\mu t}\varepsilon_{0}\rangle /\langle \varepsilon_{0}\rangle ,\\ k_{\text{sc}}=\langle \varepsilon_{0}\rangle /\langle \varepsilon_{0}+\varepsilon_{s}\rangle ,\\ k_{\text{wall}}=k_{\text{at}}k_{\text{sc}}=\langle e^{+\mu t}\varepsilon_{0}\rangle /\langle \varepsilon_{0}+\varepsilon_{s}\rangle . \end{array}$$ (7)

In Eq. (7)*k*_sc_ gives the fractional contribution to the energy deposited in the cavity gas from primary photons, and *k*_at_ corrects for the attenuation of the primary photons.

Calculations were done for the six NBS-NIST chambers used in our standard measurements. The chambers were modeled as perfect spherical shells of graphite surrounding dry air at 22 °C, 101.325 kPa (i.e., no internal electrode or external stem). Geometrical parameters used for the chambers are given in Table 3. The Spencer-Attix cut-off energy Δ listed in Table 3, to be used later, is the energy of an electron whose practical range in air is equal to the mean chord length through the cavity. For the spherical chambers, the mean chord length is 4*r*/3, where *r* is cavity radius. The practical range has been assumed to be 0.84 of the csda^1^ range for air (to approximately account for multiple-elastic-scattering detours), and the range-energy data in [15] for dry air (at 22 °C) has been used to estimate the csda range.

The chambers were assumed to exist in vacuum and to be irradiated by a parallel^2^ beam of gamma rays whose circular cross section was of a diameter equal to the outside diameter of the chamber. In order to produce results that could be used for arbitrary beam spectra, calculations for each chamber were done for mono-energetic photon beams with energies (1.33, 1.17, 1.0, 0.8, 0.66166, 0.5, 0.4, 0.3, 0.2, 0.15, 0.1, 0.08, 0.06, 0.05, 0.04, 0.03, and 0.02) MeV. The length of the secondary-electron “steps” in both graphite and air were chosen such that the electron loses an average of about 1.7 % of its energy per step and suffers deflections whose mean cosines are no smaller than 0.96.

Samples of 10^7^ incident primary photons were used for incident energies from 1.33 MeV to 0.4 MeV, 1.5 × 10^7^ for energies of 0.3 MeV and 0.2 MeV, and 2 × 10^7^ for energies of 0.15 MeV and below. At least 10 % to 20 % of the incident photons interact in the chamber^3^, depending on the incident energy and chamber dimensions. The increase in sample size for the lower incident energies was to compensate, at least partially, for the reduced contribution from the low-energy secondary electrons produced in the graphite wall penetrating into the cavity. All photon and electron histories were followed until their energy fell below 10 keV. The results for the energy deposited in the chamber air cavities had relative statistical standard deviations of 0.2 % to 0.6 %. However, because the wall-correction factors (and their components) for a particular chamber are evaluated as a ratio of correlated results, the relative statistical standard deviations are only about 0.05 % to 0.1 %.

## 3. Calculated Wall Corrections for Monoenergetic Photons

**Fig. 2:**
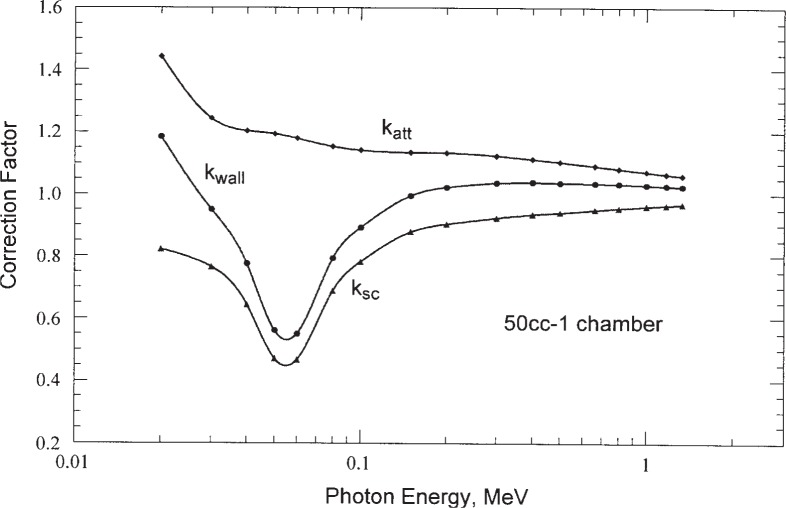
Components of the calculated wall correction for the NBS-NIST 50cc-1 standard chamber. The points are from Monte Carlo calculations for monoenergetic, parallel beams of photons; the curves are natural-cubic-spline fits to the data.

**Fig. 3:**
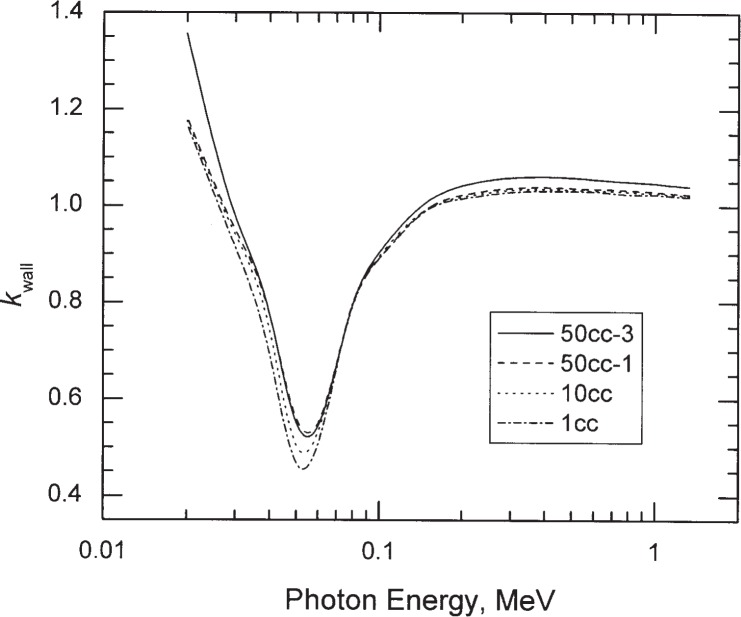
Wall corrections calculated for monoenergetic, parallel beams of photons. Results from the cubic-spline fits of the Monte Carlo data are shown for four of the NBS-NIST standard chambers.

The calculated *k*_wall_ and its components *k*_at_ and *k*_sc_ are plotted as a function of photon energy in Fig. 2 for the 50 cc-1 chamber. Table 4 gives the calculated values of *k*_wall_ as a function of incident photon energy for all of the chambers considered. Curves of *k*_wall_ vs incident photon energy are plotted in Fig. 3 for selected chambers to help illustrate differences due to changes in geometry.

These results are compared with those from test calculations with the MCNP4C code [18] for the cases of 1.25 MeV and 0.662 MeV mono-energetic photon beams incident on the chambers. For these comparisons, MCNP4C was run using identical input geometry but somewhat cruder electron-transport steps (the default choice). The comparison is presented in Table 5, which generally shows agreement to within the combined statistical uncertainties of the results obtained with the two codes (these Type A uncertainties are for a coverage factor of unity, i.e., estimated to correspond to a 67 % confidence level). Although perhaps not surprising, as the codes have similar electron-transport physics, the good agreement does tend to validate the independent code changes required to effect the correlated-sampling scheme outlined above.

## 4. Assumed Photon Spectra for NIST Gamma-Ray Sources

**Fig. 4:**
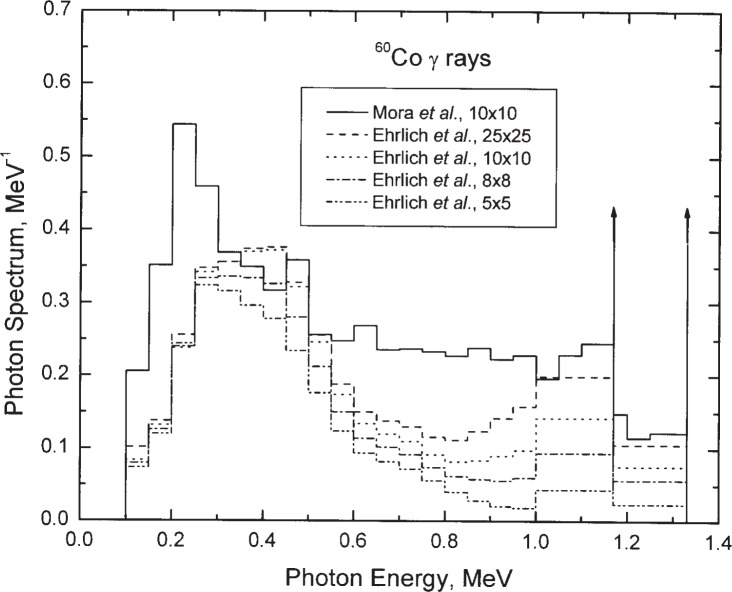
Incident photon spectra assumed for NBS-NIST therapy-level ^60^Co calibrating beams. The legend gives nominal sizes of square fields (in cm) by Ehrlich et al. [19] for a SSD of 80 cm and by Mora et al. [20] for a SSD of 100 cm. The vertical arrows indicate δ-functions at the photon energies 1.17 MeV and 1.33 MeV. The spectra are normalized to unit area.

Our final results require integration of monoenergetic results over relevant photon-fluence spectra for our gamma-ray beams. Only somewhat limited information is generally available. Spectra for ^60^Co gamma-ray fields can be found in Ehrlich et al. [19] who measured NBS spectra from the Eldorado Super G unit with the AECL G754^4^ variable collimator (in Room B034 of the NIST Bldg. 245). These spectra are assumed applicable to the similar Theratron Model F unit with the parallel-side, variable collimator (in our Room B036) also used for calibrations. The relevant spectra in Ehrlich et al. are listed for collimator settings used to produce a square field at a source-to-surface distance (SSD) of 80 cm. The reported spectra for 5 cm × 5 cm, 8 cm × 8 cm, 10 cm × 10 cm, and 2 cm × 25 cm fields have scattered-photon continua representing 14.1 %, 17.6 %, 20.0 %, and 24.0 % of the total number of incident photons, respectively. The NIST calibration fields are now at an SSD of 100 cm and, for test purposes, 150 cm, so the 8 cm × 8 cm and the 10 cm × 10 cm fields would seem perhaps most relevant. However, more recent Monte Carlo calculations of photon-fluence spectra [20, 21] consistently suggest scatter contributions of from 28 % to about 35 % for square fields of sides 5 cm to 25 cm measured at an SSD of 100 cm. Therefore, the Mora et al. spectrum [20] for a 10 cm × 10 cm at an SSD of 100 cm was included, with which the spectrum for the similar field from the independent Monte Carlo calculations of Smilowitz et al. [21] shows very good agreement. The assumed ^60^Co spectra are shown in Fig. 4. In addition, simple line spectra were also considered: monoenergetic 1.25 MeV photons, and equal-probability 1.17 MeV and 1.33 MeV photons. Thus, the spectra considered range from 0 % scatter to 35 % scatter.

**Fig. 5:**
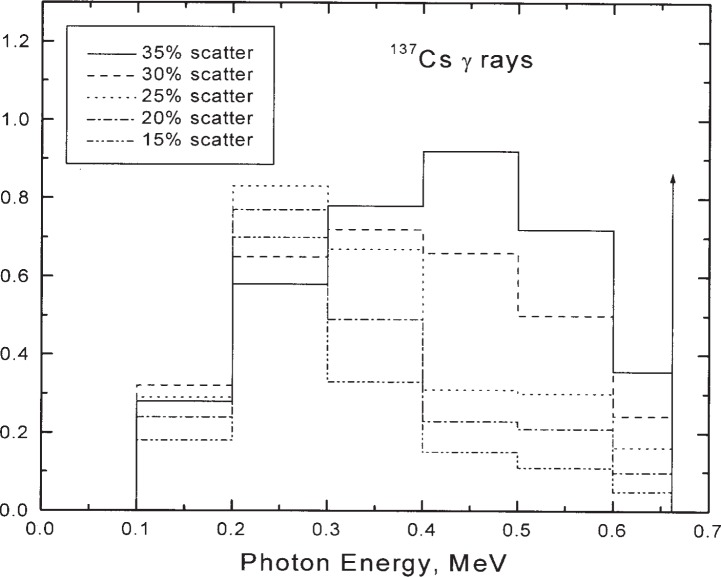
Incident photon spectra assumed for NBS-NIST ^137^Co calibrating beams. The legend gives the scatter contribution of the spectra derived from results of Costrell [22]. The vertical arrow indicates a δ -function at the photon energy 0.662 MeV. The spectra are normalized to unit area.

Spectra for ^137^Cs gamma-ray beams are given by Costrell [22], measured for eight different source geometries. For the purposes of this report, his spectra were combined (when similar) and adjusted to form five spectra with scattered-photon contributions of 15 %, 20 %, 25 %, 30 %, and 35 %, in addition to a single-line spectrum of monoenergetic 0.662 MeV photons. The ^137^Cs spectra are shown in Fig. 5.

The low-dose-rate ^192^Ir brachytherapy seed source calibrated at NBS/NIST is in the form of a right-circular cylinder of height 3 mm, composed of a 0.1 mm diameter Ir (30 %)-Pt (70 %) radioactive core (density 21.73 g/cm^3^), surrounded by a 0.2 mm thick stainless-steel annulus (density 8.06 g/cm^3^). This 0.5 mm diameter cylinder is then fitted into a cylindrical nylon annular catheter whose wall is 0.15 mm thick (density 1.14 g/cm^3^). Reference air-kerma rate is determined in air at a distance of 1 m from the source axis in the plane that perpendicularly bisects the axis. The photon spectrum at the measurement point was estimated by assuming the photon-emission probabilities for ^192^Ir decay given in Table 6, and calculating the attenuated spectrum reaching the measurement point. This calculation takes into account the attenuation along all photon paths through the various materials by integrating over all source points in the cylindrical core. Decay probabilities were taken from the National Nuclear Data Center [23] and the Lund/LBNL Nuclear Data Search [24]; photon total attenuation coefficients were taken from Berger and Hubbell [25]. The hardened line spectrum at a distance of 1 m in air from the encapsulated source is given also in Table 6, and shows that the low-energy L-shell x rays with energies up to ≈14 keV are essentially absorbed completely. The mean energy per disintegration is 350.3 keV, while that at the measurement distance of 1 m is 361.4 keV. This adopted line spectrum at 1 m ignores a continuum spectrum due to bremsstrahlung production by emitted beta particles (and conversion electrons) stopped in the seed and to Compton scattering of the transmitted photons in the air. For the ratios of interest in this work, it is expected that the results are strongly governed by the line spectrum.

## 5. Comparison of Calculated Relative Response with Measured Results

**Fig. 6:**
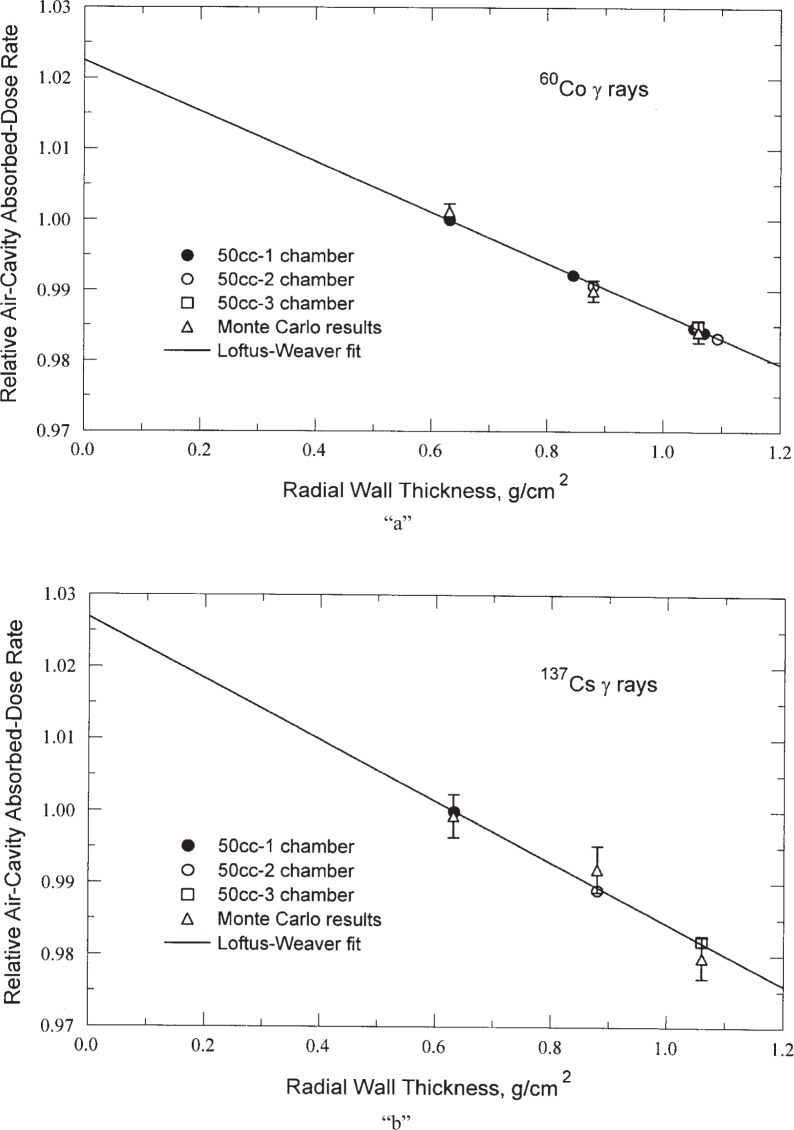
Wall-correction data of Loftus and Weaver [11] with Monte Carlo results for the 50-1, 50-2, and 50-3 chambers added for comparison. The Monte Carlo results are normalized to the same incidence fluence rate, and are based on the calculated absorbed-dose rate in the air cavity: (a) ^60^Co, assuming the Mora et al. [20] spectrum. (b) ^137^Cs, assuming the 30 % scatter spectrum. The error bars represent the estimated relative standard deviations for the ratios of the Monte Carlo results.

The calculated absorbed-dose rates in the air cavity, as a function of the wall thickness of the 50-series chambers is shown in Fig. 6, where they are compared with the experimental data that Loftus and Weaver used in the extrapolation (*k*_extrap_) for their experimental wall correction. Although agreement is good, the relatively large uncertainties of the Monte Carlo results preclude a more definitive confirmation of the calculations; additional calculations with larger numbers of Monte Carlo histories could be done to further address this point.

## 6. Ratios of Photon Mass Energy-Absorption Coefficients

Integrating the incident fluence spectra over the relevant photon mass energy-absorption coefficients from Seltzer [26] and Hubbell and Seltzer [27], the air-to-carbon ratios obtained for the ^60^Co, ^137^Cs and ^192^Ir sources are given in Table 7. For each of these radionuclides, the results obtained for the various spectra assumed in this report vary by only a maximum of 0.02 % from the value given for that radionuclide in Table 7. The very small differences from the NBS-NIST 1986 values as shown in Table 7 is within that due to round-off from the use of three significant figures in the adjustment factors given in Table 1. Note, however, that values for the photon mass energy-absorption coefficients used here [26] differ in significant respects from those used for the 1986 ratios [13] and that the assumed incident photon spectra are also no doubt different; clearly much of these differences disappear in the ratio of mass energy-absorption coefficients for two materials of not too dissimilar composition. These results suggest that a conservative estimate for the relative standard uncertainty of (μ¯enρ)graphiteair is about 0.06%.

The radiative losses summarized in the parameter $\bar{g}$ are evaluated for the determination of the photon mass energy-absorption coefficient. For the data used here [26], the radiative yields include a small correction that takes into account the fluctuations in energy-loss suffered by an electron in the course of slowing down, in contrast to the usual assumption of the continuous-slowing-down approximation. Although the effect on the relevant quantity, $1-\bar{g}$, is quite small, the results obtained for the various assumed spectra are listed in Table 8. The $\bar{g}$ values adopted here are 0.0033 for ^60^Co, 0.0018 for ^137^Cs and 0.0012 for ^192^Ir. The corresponding values of $1-\bar{g}$ are then 0.9967 for ^60^Co, 0.9982 for ^137^Cs and 0.9988 for ^192^Ir, all with an estimated relative standard uncertainty of 0.02 %.

## 7. Stopping-Power Ratios

**Fig. 7:**
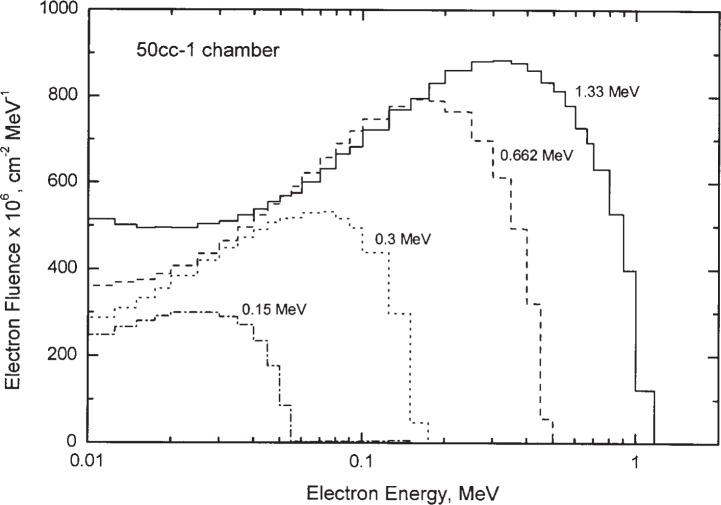
Spectra of electron fluence for the NIST 50cc-1 chamber in monoenergetic photon fields. Results are from Monte Carlo calculations for the chamber in a parallel beam, normalized to one incident photon. The histograms are the electron-fluence spectra scored in the air cavity, and are shown for incident photon energies of 1.33 MeV, 0.662 MeV, 0.3 MeV, and 0.15 MeV.

Electron fluence spectra *Φ* (*T*), as a function of electron kinetic energy *T*, in the air cavity and in the graphite walls, including all electrons set in motion by primary and scattered photons, were obtained in all the calculations. Examples of the calculated electron fluence spectra in the air cavity are illustrated in Fig. 7 for monoenergetic photons incident on the 50cc-1 chamber. Graphite-to-air stopping-power ratios were then evaluated according to Spencer-Attix cavity theory [28] with the Nahum [29] track-end term:

(S¯ρ)airgraphite=∫ΔT01ρLgraphite(T,Δ)Φ(T)dT+1ρSgraphite(Δ)Φ(Δ)Δ∫ΔT01ρLair(T,Δ)Φ(T)dT+1ρSair(Δ)Φ(Δ)Δ, (8)

where *T*_0_ is the kinetic energy of the most energetic electron set in motion, *L*(*T*,*Δ*) is the restricted electronic stopping power [15], *S* is the unrestricted electronic stopping power [15], and *Δ* is the appropriate cut-off energy for each chamber as described in Sec. 2 and listed in Table 3. The stopping-power ratio defined in Eq. (8) is essentially the ratio of absorbed doses in graphite and in air calculated from the electron-fluence spectrum in the cavity. The calculation of electronic stopping powers for a medium is straightforward [15] once parameters are chosen that define two non-trivial terms in the Bethe stopping-power formula: the mean excitation energy, *I*, and the density-effect correction, *δ*. For a distributed incident photon spectrum, mono-energetic results for the numerator and the denominator in eq. (8) are each integrated over the incident photon spectrum before the ratio is taken. Results for the various chambers and assumed spectra are summarized in Table 9, based on currently recommended values of *I* and parameters that determine *δ*.

Graphite is not a simple homogeneous material. It consists of weakly bound sheets of carbon crystals with a crystallite density of approximately 2.265 g/cm^3^. Bulk graphite is porous and can be assumed to consist of these carbon crystals and voids (air). If bulk graphite is treated as a simple mixture of carbon crystals and air, then a bulk density of 1.73 g/cm^3^ would imply a fraction by weight for air of 0.0164 %. The ICRU [15] has recommended the use of the bulk density for a material in calculation of the density effect, but—for purposes of illustration—considers also treating inhomogeneous materials as a mixture. Applied to the case of graphite, the mixture approach gives values of the electronic stopping power that are the same to four significant figures as those for pure graphite with the crystallite density of 2.265 g/cm^3^. This is consistent with the suggestion of Rogers et al. [30] who find better agreement with the measured energy loss of 6 MeV to 28 MeV electrons in graphite when they use a density of 2.26 g/cm^3^ instead of 1.70 g/cm^3^ for the calculation of the density-effect correction.

The value recommended by the International Commission on Radiation Units and Measurements (ICRU) [15] for the mean excitation of carbon (graphite) is 78.0 ± 7.0 eV. Since that critical evaluation, a value of 86.9 ± 1.7 eV has been extracted by Bichsel and Hiraoka [31] from their measurements of the energy loss of 70 MeV protons. The question about possible new recommended values of the density and the mean excitation energy for graphite is being considered by the CCRI(I) and the ICRU. The effects on the stopping-power ratios due to some possible changes of graphite parameters are illustrated in Table 10, considering the change of density from 1.73 g/cm^3^ to 2.265 g/cm^3^ and the change of mean excitation energy from 78.0 eV to the Bichsel-Hiraoka value of 86.9 eV, along with an intermediate value^5^ of 82.4 eV.

As can be seen in Table 10, the change of the assumed density of graphite to the crystallite density of 2.265 g/cm^3^ in the calculation of the density effect lowers our calculated stopping-power ratios by ≈0.21 % for ^60^Co, ≈0.11 % for ^137^Cs, and ≈0.06 % for ^192^Ir. The change in the mean excitation energy for graphite can have a significantly larger effect, possibly an additional reduction of about from 0.7 % to 1.3 % for ^60^Co, from 0.7 % to 1.5 % for ^137^Cs, and from 0.8 % to 1.5 % for ^192^Ir. However, until there is international consensus on a recommended new value for the mean excitation and on a different method to evaluate the density effect for graphite, NIST will continue to use the current value (*I*_graphite_ = 78.0 eV, density of 1.73 g/cm^3^) in calculations of factors used in our standards. The differences in the graphite-to-air stopping-power ratios from our calculations using the current standard values, compared to the modified Loftus-Weaver values given in Table 2, are listed in Table 11. If the stopping-power ratios are evaluated using the electron-fluence spectra established in the graphite wall^6^ rather than the air cavity, the new ratios would be reduced by only from 0.01 % to 0 04 %.

Anticipating a re-evaluation of *I*_graphite_ and density-effect parameters, and international consensus on their values, NIST is temporarily increasing the stated uncertainty of the stopping-power ratio to accommodate possible future changes. Therefore, relative standard uncertainties estimated to be 0.57 % for ^60^Co, 0.62 % for ^137^Cs, and 0.72 % for ^192^Ir will be used for the stopping-power ratios until agreement on new stopping-power parameters has been established.

## 8. Humidity Corrections

The vented ionization chambers are filled with ambient air, which in usual laboratory conditions contain a quantity of water vapor. The correction for the influence of humid air (i.e., to correct the measurement to that of kerma for dry air for which the analysis of Bragg-Gray theory is routinely done) is given [16] by

$$k_{h}=\frac{W_{\text{humid air}}}{W_{\text{dry air}}}\frac{\rho_{\text{dry air}}{\bar{\left(S/\rho \right)}}_{\text{dry air}}}{\rho_{\text{humid air}}{\bar{\left(S/\rho \right)}}_{\text{humid air}}}.$$ (9)

**Fig. 8:**
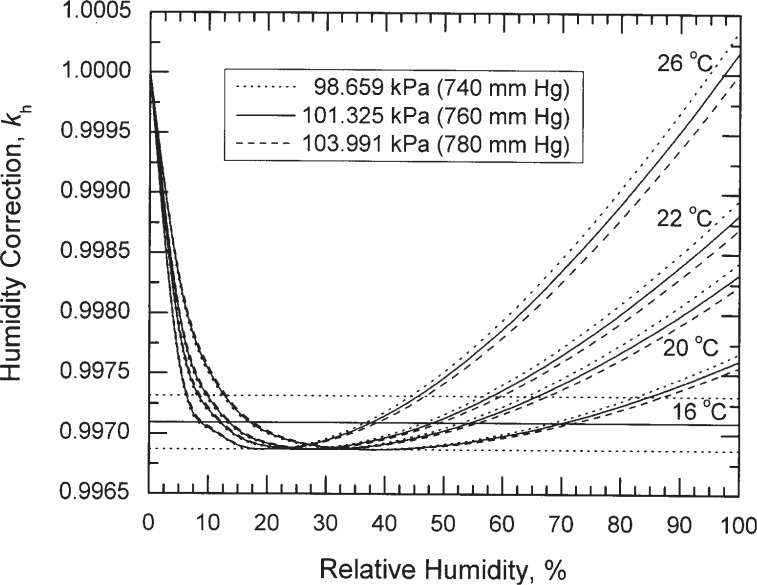
Humidity corrections for the NBS-NIST graphite-wall air-ionization cavity chambers irradiated by ^60^Co, ^137^Cs and ^192^Ir gamma rays. The results are insensitive to the assumed energy spectra and the chamber dimensions.

The density of humid air was calculated using the equations of Giacomo^7^ [32], which take into account the small CO_2_ content, the compressibility of the air-water-vapor mixture, and the enhancement factor (that expresses the fact that the effective saturation vapor pressure of water in air is greater than the saturation vapor pressure of pure vapor phase over a plane of pure liquid water). The variation of *W*_humid air_/*W*_dry air_ as a function of the partial pressure of water vapor was taken from the curve in ICRU [16] based on the results of Niatel [35]. In general, the result for *k*_h_ is a complicated function of temperature, pressure, relative humidity, and secondary-electron spectrum (hence of the primary photon spectrum and the geometrical details of the chamber). The electron fluence spectra in the chamber air cavities used to calculate the (S¯ρ)airgraphitestopping-power ratios have been used also to calculate the (S¯ρ)humid airdry air stopping-power ratios for the humidity correction. Our results show negligible dependence on the assumed incident photon spectrum (for ^60^Co, ^137^Cs, or ^192^Ir sources) and on the geometric variations among the NBS-NIST standard detectors, so that the humidity correction becomes a function of only relative humidity, temperature, and pressure. Humidity corrections are plotted in Fig. 8 for the range of conditions considered in our calculations.

It is perhaps helpful to present results simply as a function of the fraction by weight of water vapor assumed in the humid air. The resultant factors are listed in Table 12, covering the range of conditions likely to be of interest in the laboratory. The relationship between the fraction by weight of water vapor and ambient atmospheric conditions is illustrated in Table 13. As can be seen, laboratory conditions typically correspond to a range of water-vapor content of from about 0.25 % to 1.5 % by weight. Calibration conditions in the NIST laboratories are at temperatures between 22 °C and 24 °C, atmospheric pressures 98.66 kPa (740 mm Hg) and 103.99 kPa (780 mm Hg), and relative humidities between 20 % and 50 %. For these conditions, one would predict a value for the humidity correction *k*_h_ of from 0.9969 to 0.9973, with a mean reference value of 0.9971 (see the horizontal lines in Fig. 8). This reference value is nearly the same as that adopted in 1986, but applies to a somewhat more restricted range than that earlier indicated (which was for relative humidities from 10 % to 70 %; temperatures and pressures unstated).

For NIST conditions, the value of 0.9971 for *k*_h_ has a relative standard uncertainty estimated to be about 0.06 %, due mainly to the uncertainty of *W*_humid air_/*W*_dry air_.

## 9. Wall Corrections Calculated for Assumed Spectra

### 9.1. ^60^Co, ^137^Cs, and ^192^Ir Beams

The Monte Carlo wall correction is evaluated as the ratio of the “unattenuated first-collision” energy deposition in the cavity to that from all particles (the “usual” total energy deposition), i.e., $k_{\text{wall}}^{\text{MC}}=\langle e^{+ìz}\varepsilon_{0}\rangle /\langle \varepsilon \rangle$. For a distributed spectrum, numerator and denominator are each evaluated through the appropriate integral over the spectrum before the ratio is obtained. The Monte Carlo $k_{\text{wall}}^{\text{MC}}$ values for the NBS-NIST chambers are listed in

Table 14 for our assumed spectra.

### 9.2. Possible Deviations From Cavity Theory

Cavity theory is based on the assumption that photons interact only in the surrounding medium, with the gas-filled cavity representing a negligible perturbation. For realistic chambers with cavities of significant volume and for primary photons of lower energies, for which the probability of interacting in the gas might be non-negligible, the use of Bragg-Gray and Spencer-Attix cavity theory has been questioned. With particular concern for ^192^Ir, Borg et al. [36] studied various aspects of Spencer-Attix cavity theory using extensive Monte Carlo calculations and concluded that the theory can be applied to ^192^Ir with an accuracy of about 0.1 % to 0.2 %. We have looked at deviations from Spencer-Attix theory using the correlated-sampling scheme in which results were scored separately for primary photons first interacting in the wall and first interacting in the cavity air. In this case, we assume a modified relationship governing the air kerma:

$$\begin{array}{l} {K^{\prime }}_{\text{air}}\approx \frac{1}{V\rho_{\text{air}}}\frac{\left(W_{\text{air}}/e\right)}{1-\bar{g}}\left[\frac{{\bar{\left(S/\rho \right)}}_{\text{graphite}}}{{\bar{\left(S/\rho \right)}}_{\text{air}}}\frac{{\bar{\left(\mu_{\text{en}}/\rho \right)}}_{\text{air}}}{{\bar{\left(\mu_{\text{en}}/\rho \right)}}_{\text{graphite}}}Q_{1}k_{\text{wall}}^{\left(1\right)}+Q_{2}k_{\text{wall}}^{\left(2\right)}\right]\prod_{i\neq \text{wall}}k_{i},\\ {K^{\prime }}_{\text{air}}\approx \frac{Q_{\text{air}}}{V\rho_{\text{air}}}\frac{\left(W_{\text{air}}/e\right)}{1-\bar{g}}\left[\frac{{\bar{\left(S/\rho \right)}}_{\text{graphite}}}{{\bar{\left(S/\rho \right)}}_{\text{air}}}\frac{{\bar{\left(\mu_{\text{en}}/\rho \right)}}_{\text{air}}}{{\bar{\left(\mu_{\text{en}}/\rho \right)}}_{\text{graphite}}}(1-\alpha )k_{\text{wall}}^{\left(1\right)}+\alpha k_{\text{wall}}^{\left(2\right)}\right]\prod_{i\neq \text{wall}}k_{i}, \end{array}$$ (10)

where *Q*_1_ is the ionization in the cavity air and $k_{\text{wall}}^{\left(1\right)}={\langle e^{+\mu z}\varepsilon_{0}\rangle }^{\left(1\right)}/{\langle \varepsilon \rangle }^{\left(1\right)}$ the wall correction for the primary photons first interacting *in the graphite wall*, and *Q*_2_ is the ionization in the cavity air and $k_{\text{wall}}^{\left(2\right)}={\langle e^{+\mu z}\varepsilon_{0}\rangle }^{\left(2\right)}/{\langle \varepsilon \rangle }^{\left(2\right)}$ the wall correction for primary photons first interacting *in the cavity air*; *α* is simply *Q*_2_/(*Q*_1_+*Q*_2_) = *Q*_2_/*Q*_air_, the fraction of the cavity ionization produced by primary photons first interacting in the cavity air. Then, by introducing a cavity-theory correction factor, *k*_cav_, and equating *K*′_air_ = *k*_cav_*K*_air_, we define *k*_cav_ as the ratio of Eqs. (10) and (6):

$$k_{\text{cav}}=\frac{(1-\alpha )k_{\text{wall}}^{\left(1\right)}+\alpha k_{\text{wall}}^{\left(2\right)}\frac{{\bar{\left(S/\rho \right)}}_{\text{air}}}{{\bar{\left(S/\rho \right)}}_{\text{graphite}}}\frac{{\bar{\left(\mu_{\text{en}}/\rho \right)}}_{\text{graphite}}}{{\bar{\left(\mu_{\text{en}}/\rho \right)}}_{\text{air}}}}{k_{\text{wall}}^{\text{MC}}},$$ (11)

where $k_{\text{wall}}^{\text{MC}}$ is the “standard” wall correction, calculated without separating out first interactions in the cavity gas by primary photons. Note that all factors in Eq. (11) are the results of integrating the appropriate quantities over the assumed spectrum.

The relevant chamber for the ^192^Ir-source measurements is the 50cc-1, the only chamber for which this dual scoring was done. The results for our assumed spectra are listed in Table 15. As can be seen in Table 15, our calculated deviations from cavity theory are negligible for ^60^Co and for ^137^Cs. Our predicted deviation for the ^192^Ir seed source is 0.15 %, in very good agreement with the conclusion (0.1 % to 0.2 %) of Borg et al. [36] from their independent investigations.

## 10. Adopted Wall Corrections

The small differences in the wall corrections calculated for different assumed spectra are numerically significant because they are the result of integrations over the same monoenergetic results. However, as can be seen in Table 14, the calculated wall corrections are rather insensitive to the assumed spectra, varying only by about 0.1 % among assumed spectra that include a significant scatter contribution and by no more than 0.2 % even if the monoenergetic 1.25 MeV line is included among the ^60^Co spectra. The adopted wall corrections and their differences from the Loftus-Weaver adjusted linear-extrapolation $k_{\text{wall}}^{\exp}$ values are given in Table 16.

Based on a statistical relative standard deviation of 0.1 %, a remaining spectrum relative uncertainty of ≈0.1 %, and a modeling relative uncertainty of ≈0.1 %, the relative standard uncertainty of the adopted wall corrections is estimated to be about 0.17 %.

## 11. Implications for NIST Exposure and Air-Kerma Primary Standards

Earlier recommendations of Bielajew and Rogers [37], based on EGS Monte Carlo calculations for the NBS-NIST chambers, suggest an increase in wall corrections of 0.89 %, 0.81 %, 0.92 %, 0.84 %, 0.97 %, and 0.94 % for the 1cc, 10cc, 30cc 50cc-1, 50cc-2, and 50cc-3 chambers, respectively. More recently, using EGSnrc Monte Carlo calculations, Rogers and Treurniet [8] suggest an increase in *k*_wall_ for a ^60^Co beam of 1.00 % and 0.96 %, for the NIST 30cc and 50cc-1 chambers^8^, respectively. The values from these two calculations agree with each other and with the NIST results given here to within about 0.1 %, a difference no larger than the statistical uncertainty estimated for the NIST results. This level of agreement, along with that between our ACCEPT and MCNP results indicated in Table 5, suggest that calculations of wall corrections as ratios of correlated results are rather insensitive to differences among the transport algorithms and radiation-interaction data used in current Monte Carlo codes.

Our present results, given in Tables 7, 8, 11, 12 and 16, lead to the following changes in NIST air-kerma standards: +0.87 % to +1.11 % (depending on the chamber) for ^60^Co, +0.64 % to +1.07 % (depending on the chamber) for ^137^Cs, and –0.06 % for the single chamber used in the measurement of the standardized ^192^Ir source. NIST has a number of fixed gamma-ray sources used to calibrate instruments in terms of air-kerma; these are listed in Table 17. The two ^60^Co vertical beams, in Rooms B034 and B036, are being re-measured with an appropriate subset of standard chambers. Small changes are expected as a result of changing to a commonly accepted field size. The results of these measurements will first be analyzed using the current 1986 values of the Bragg-Gray and correction factors, to isolate the effects of geometry and measurement-technique changes. The primary standard will then be adjusted to reflect the adoption of the new factors described in this report. For the remaining beams, the numerical changes in the adopted factors will be used in the switch to the new standard until a program of re-measurement can be completed. This will be based on the following scheme.

Over the last few decades, NBS-NIST primary air-kerma standards have been based on the historical weighted mean of results given by Loftus and Weaver [11]. Their Table 13 gave the factor required to bring the measurement for each chamber into agreement with the weighted-mean value. Those factors have continued to be used, applied to measurements involving only a subset of the original suite of chambers. Using the results calculated here, a new relationship can be established. Loftus and Weaver determined the weighted-mean exposure rate as

$${\dot{X}}_{\text{std}}=\sum_{i}\omega_{i}{\dot{X}}_{i},$$ (12)

where the relative weight *ω_i_* is based on the measurement uncertainty for the *i*th chamber. The correction factor for the *j*th chamber is

$${\;}_{j}k_{\text{std}}=\frac{\sum_{i}\omega_{i}{\dot{X}}_{i}}{{\dot{X}}_{j}}=\frac{{\dot{X}}_{\text{std}}}{{\dot{X}}_{j}}.$$ (13)

Introducing the chamber-specific changes *R_i_* from the Monte Carlo calculations, mainly due to the wall corrections,

$${{\dot{X}}^{\prime }}_{\text{std}}=\sum_{i}\omega_{i}{{\dot{X}}^{\prime }}_{i}=\sum_{i}\omega_{i}R_{i}{\dot{X}}_{i}=\sum_{i}R_{i}\omega_{i}\frac{{\dot{X}}_{\text{std}}}{{\;}_{i}k_{\text{std}}},$$ (14)

and

$$\bar{R}=\frac{{{\dot{X}}^{\prime }}_{\text{std}}}{{\dot{X}}_{\text{std}}}=\sum_{i}R_{i}\frac{\omega_{i}}{{\;}_{i}k_{\text{std}}},$$ (15)

is then the final change in the primary standard for exposure (and air-kerma) rate. This change determines the new relationship of the individual chambers to the new standard:

$${\;}_{j}{k^{\prime }}_{\text{std}}=\frac{{{\dot{X}}^{\prime }}_{\text{std}\;}}{{X^{\prime }}_{j}}=\frac{\bar{R}{\dot{X}}_{\text{std}}}{R_{j}{\dot{X}}_{j}}=\frac{\bar{R}}{R_{j}}{\;}_{j}{k^{\prime }}_{\text{std}}.$$ (16)

Results for the factors are given in Table 18. Two evaluations were done: (a) using only the changes in the wall corrections given in Table 16, and (b) including also the small changes in the photon mass energy-absorption ratio (from Table 7), in ${\left(1-\bar{g}\right)}^{-1}$ (from Table 8), in the electron mass stopping-power ratios (from Table 11), and in the humidity correction (+0.01 %).

In earlier work, Bielajew and Rogers [37] and Rogers and Treurniet [8] employed their Monte Carlo calculations done for many of the chambers used by the major metrology institutions to assess the new relationship among standards after adoption of new correction factors (primarily *k*_wall_). From their calculations, Rogers and Treurniet^9^ [8] suggest a shift of the BIPM ^60^Co air-kerma standard (the international reference value) by the factor 1.0046. Accepting their results for the BIPM standard and a change by the factor 1.0088 from Table 18 as representative for the NIST standard, the ratio of the current NIST standard to the current BIPM standard for ^60^Co air-kerma of ${\left(\frac{{\dot{K}}_{\text{NIST}}}{{\dot{K}}_{\text{BIPM}}}\right)}_{\text{current}}=0.9980\;\text{would change to}{\left(\frac{{\dot{K}}_{\text{NIST}}}{{\dot{K}}_{\text{BIPM}}}\right)}_{\text{revised}}=1.0022$, without the inclusion of the small changes in electron stopping-power and photon energy-absorption ratios that would presumably affect both standards. Thus, the NIST-BIPM level of agreement for ^60^Co air-kerma can be expected to remain at about the 0.2 % level with the adoption of our new wall corrections; only the sign would change.

## Figures and Tables

**Table 1:** Changes made in 1986 to correction factors for NBS-NIST primary air-kerma standards for gamma rays

Quantity	Multiply earlier values^a^ by:		
	^60^Co	^137^Cs	^192^Ir
Humidity	0.997	0.997	0.997
(μ¯enρ)graphiteair	0.999	1.000	1.000
(S¯ρ)airgraphite	0.993	0.995	0.996

Total	0.989	0.992	0.993

a For ^60^Co and ^137^Cs, see Loftus and Weaver [11]; for ^192^Ir, see Loftus [10].

**Table 2:** Summary of pertinent correction factors, as modified 1 January 1986, for NBS-NIST primary-standard graphite-wall ionization chambers

Chamber	$k_{\text{wall}}^{\exp}$	(S¯ρ)airgraphite	(μ¯enρ)graphiteair
^60^Co			

1cc	1.0117	0.9999	0.9985
10cc	1.0165	0.9994	0.9985
30cc	1.0169	0.9992	0.9985
50cc-1	1.0176	0.9991	0.9985
50cc-2	1.0267	0.9991	0.9985
50cc-3	1.0335	0.9991	0.9985

^137^Cs			

1cc	1.0189	1.0092	0.9997
10cc	1.0250	1.0087	0.9997
30cc	1.0239	1.0084	0.9997
50cc-1	1.0262	1.0082	0.9997
50cc-2	1.0374	1.0082	0.9997
50cc-3	1.0457	1.0082	0.9997

^192^Ir			

50cc-1	1.033	1.015	1.002

**Table 3:** Dimensions of the NBS-NIST spherical graphite ionization chambers

Chamber (cm)	Outside diameter (cm)	Inside diameter (cm)	Wall thickness (cm)	Graphite density (g/cm^3^)	Mean chord length (cm)	Cut-off energy Δ (keV)
1cc	2.065	1.270	0.398	1.73	0.847	22.5
10cc	3.428	2.677	0.376	1.72	1.785	34.4
30cc	4.607	3.857	0.375	1.74	2.571	42.3
50cc-1	5.340	4.610	0.365	1.73	3.073	46.8
50cc-2	5.580	4.563	0.509	1.73	3.042	46.6
50cc-3	5.800	4.574	0.613	1.73	3.049	46.6

**Table 4:** Calculated wall corrections, *k*_wall_, for the NBS-NIST spherical graphite ionization chambers

Photon Energy, MeV	Chamber					
	50cc-3	50cc-2	50cc-1	30cc	10cc	1cc
1.3300	1.0401	1.0327	1.0241	1.0238	1.0211	1.0188
1.1700	1.0428	1.0373	1.0265	1.0261	1.0241	1.0208
1.0000	1.0467	1.0397	1.0297	1.0292	1.0264	1.0237
0.8000	1.0501	1.0431	1.0329	1.0321	1.0295	1.0243
0.6617	1.0533	1.0469	1.0349	1.0348	1.0314	1.0286
0.5000	1.0585	1.0489	1.0367	1.0369	1.0335	1.0309
0.4000	1.0602	1.0479	1.0386	1.0374	1.0349	1.0312
0.3000	1.0580	1.0500	1.0364	1.0344	1.0326	1.0284
0.2000	1.0413	1.0339	1.0223	1.0230	1.0185	1.0148
0.1500	1.0093	1.0023	0.9949	0.9897	0.9949	0.9924
0.1000	0.9024	0.8997	0.8906	0.8900	0.8944	0.8910
0.0800	0.7927	0.7885	0.7920	0.7898	0.7893	0.7958
0.0600	0.5432	0.5452	0.5491	0.5413	0.5308	0.5057
0.0500	0.5513	0.5533	0.5607	0.5432	0.5042	0.4674
0.0400	0.7761	0.7729	0.7741	0.7636	0.7431	0.6926
0.0300	0.9780	0.9691	0.9497	0.9473	0.9397	0.9136
0.0200	1.3562	1.2818	1.1840	1.1857	1.1766	1.1682

**Table 5:** Values of *k*_wall_ calculated with the ACCEPT/ITS3 and the MCNP4C Monte Carlo codes. The ACCEPT calculations are based on a 1.7 % average energy loss per electron step; the MCNP4C calculations on a 2.8 % average energy loss per electron step. The statistical uncertainties shown are relative standard deviations of the means of the calculated results

Chamber	0.662 MeV photons		1.25 MeV photons	
	ACCEPT	MCNP4C	ACCEPT	MCNP4C
1cc	1.0286 ± (0.08 %)	1.0298 ± (0.05 %)	1.0197 ± (0.04 %)	1.0202 ± (0.07 %)
10cc	1.0314 ± (0.06 %)	1.0327 ± (0.05 %)	1.0226 ± (0.03 %)	1.0230 ± (0.03 %)
30cc	1.0348 ± (0.07 %)	1.0351 ± (0.04 %)	1.0249 ± (0.03 %)	1.0252 ± (0.03 %)
50cc-1	1.0349 ± (0.05 %)	1.0353 ± (0.06 %)	1.0252 ± (0.03 %)	1.0261 ± (0.03 %)
50cc-2	1.0469 ± (0.08 %)	1.0475 ± (0.10 %)	1.0351 ± (0.04 %)	1.0354 ± (0.05 %)
50cc-3	1.0533 ± (0.09 %)	1.0551 ± (0.09 %)	1.0413 ± (0.04 %)	1.0415 ± (0.04 %)

**Table 6:** ^192^Ir photon line spectra, including photons with energies greater than 10 keV. The data for energies below 100 keV are for the Pt and Os x rays emitted in the decay of ^192^Ir; x rays with energies up to 14 keV make a negligible contribution to final results at 1 m, given for the seed in catheter

Energy, keV	Relative probability per decay^a^	Relative probability at 1 m
10.176	0.000180	0.000000
10.354	0.001750	0.000000
10.511	0.000249	0.000000
10.590	0.000570	0.000000
10.820	0.000091	0.000000
10.854	0.000240	0.000000
11.071	0.005319	0.000000
11.235	0.000313	0.000000
11.242	0.001518	0.000000
11.562	0.000125	0.000000
12.096	0.000339	0.000000
12.422	0.000057	0.000000
12.500	0.000081	0.000000
12.942	0.001051	0.000000
13.271	0.000129	0.000000
13.273	0.000077	0.000000
13.361	0.000107	0.000000
61.486	0.005147	0.003225
63.000	0.008879	0.005737
65.122	0.011367	0.007636
66.831	0.019431	0.013429
71.079	0.001025	0.000753
71.414	0.001973	0.001455
71.875	0.000048	0.000036
73.363	0.000695	0.000524
73.590	0.000081	0.000061
75.368	0.002286	0.001763
75.749	0.004414	0.003418
76.233	0.000114	0.000074
77.831	0.001566	0.001041
78.073	0.000205	0.000137
110.093	0.000053	0.000039
136.343	0.000785	0.000663
201.3112	0.002027	0.001966
205.795	0.014241	0.013876
280.2	0.000069	0.000070
283.2668	0.001132	0.001153
295.957	0.123105	0.125787
308.456	0.127995	0.131221
316.507	0.354989	0.364624
329.2	0.000077	0.000080
374.4852	0.003148	0.003267
416.47	0.002861	0.002984
420.53	0.000305	0.000318
468.07	0.205118	0.214867
484.58	0.013666	0.014332
489.05	0.001892	0.001984
588.58	0.019371	0.020427
593.4	0.000180	0.000190
604.41	0.035259	0.037205
612.46	0.022820	0.024087
884.54	0.001252	0.001332
1061.48	0.000227	0.000242

a Multiply by 2.331338 for number/disintegration

**Table 7:** Air-to-graphite photon mass energy-absorption coefficient ratios from this work

Source	(μ¯enρ)graphiteair	Percent differences from NBS-NIST 1986 values
^60^Co	0.9990	+0.05
^137^Cs	0.9993	−0.04
^192^Ir	1.0016	−0.04

**Table 8:** Values of the mean fraction of the kinetic energy of electrons (and positrons) liberated by the photons that is lost in radiative processes in air

Spectrum	$\bar{g}$	Percent differences in $1-\bar{g}$ from current NIST values
^60^Co, 1.25 MeV photons	0.0035	
^60^Co, 1.17 MeV +1.33 MeV photons	0.0035	
^60^Co, Ehrlich et al., 5 × 5 cm^2^ field	0.0034	
^60^Co, Ehrlich et al., 8 × 8 cm^2^ field	0.0034	
^60^Co, Ehrlich et al., 10 × 10 cm^2^ field	0.0033	
^60^Co, Ehrlich et al., 25 × 25 cm^2^ field	0.0033	
^60^Co, Mora et al., 10 × 10 cm^2^ field	0.0033	+0.01

^137^Cs, 0.662 MeV photons	0.0019	
^137^Cs, 15 % scatter spectrum	0.0018	
^137^Cs, 20 % scatter spectrum	0.0018	
^137^Cs, 25 % scatter spectrum	0.0018	
^137^Cs, 30 % scatter spectrum	0.0018	+0.02
^137^Cs, 35 % scatter spectrum	0.0018	

^192^Ir seed, at 1 m	0.0012	+0.12

**Table 9:** Electron mass stopping-power ratios (S¯ρ)airgraphite for NBS-NIST spherical graphite cavity ionization chambers, from Monte Carlo calculations. Results are based on the parameters given in ICRU [15] for dry air at 22 °C, and 101.325 kPa, and for graphite with a density of 1.73 g/cm^3^ and a mean excitation energy of 78.0 eV

Chamber	1.25 MeV photons (0 % scatter)	1.17+1.33 MeV photons (0 %)	Ehrlich et al., 5 × 5 cm^2^ field (14.1 %)	Ehrlich et al., 8 × 8 cm^2^ field (17.6 %)	Ehrlich et al., 10 × 10 cm^2^ field (20.9 %)	Ehrlich et al., 25 × 25 cm^2^ field (24.0 %)	Mora et al., 10 × 10 cm^2^ field (32.7 %)

^60^Co							
1cc	0.9995	0.9995	1.0001	1.0003	1.0004	1.0005	1.0009
10cc	0.9991	0.9990	0.9996	0.9998	0.9999	1.0000	1.0004
30cc	0.9989	0.9989	0.9994	0.9996	0.9997	0.9998	1.0002
50cc-1	0.9988	0.9988	0.9993	0.9995	0.9996	0.9997	1.0001
50cc-2	0.9988	0.9988	0.9994	0.9995	0.9996	0.9997	1.0001
50cc-3	0.9989	0.9988	0.9994	0.9995	0.9996	0.9997	1.0001

^137^Cs						

Chamber	0.662 MeV photons	15 % scatter spectrum	20 % scatter spectrum	25 % scatter spectrum	30 % scatter spectrum	35 % scatter spectrum
1cc	1.0089	1.0092	1.0093	1.0094	1.0096	1.0097
10cc	1.0084	1.0087	1.0088	1.0089	1.0090	1.0091
30cc	1.0082	1.0084	1.0085	1.0086	1.0087	1.0088
50cc-1	1.0080	1.0083	1.0084	1.0085	1.0086	1.0087
50cc-2	1.0081	1.0083	1.0084	1.0085	1.0086	1.0087
50cc-3	1.0081	1.0083	1.0084	1.0085	1.0086	1.0087

^192^Ir	

Chamber	^192^Ir (1 m)
50cc-1	1.0116

**Table 10:** Electron mass stopping-power ratios (S¯ρ)airgraphitefor NBS-NIST spherical graphite cavity ionization chambers, from Monte Carlo calculations. Results are based on the parameters given in ICRU [15] for dry air at 22 °C, and 101.325 kPa, but assuming different combinations of the density and the mean excitation energy for graphite

Chamber	1.73 g/cm^3^ 78.0 eV	2.265 g/cm^3^ 78.0 eV	2.265 g/cm^3^ 82.4 eV	2.265 g/cm^3^ 86.9 eV
^60^Co, Mora et al. spectrum.				

1cc	1.0009	0.9988	0.9919	0.9851
10cc	1.0004	0.9983	0.9917	0.9852
30cc	1.0002	0.9980	0.9915	0.9851
50cc-1	1.0001	0.9979	0.9915	0.9852
50cc-2	1.0001	0.9980	0.9915	0.9852
50cc-3	1.0001	0.9980	0.9916	0.9853

^137^Cs, 30 % scatter spectrum				

1cc	1.0096	1.0085	1.0008	0.9932
10cc	1.0090	1.0079	1.0004	0.9931
30cc	1.0087	1.0076	1.0003	0.9932
50cc-1	1.0086	1.0075	1.0002	0.9931
50cc-2	1.0086	1.0075	1.0003	0.9931
50cc-3	1.0086	1.0075	1.0003	0.9931

^192^Ir				

50cc-1	1.0116	1.0110	1.0032	0.9957

**Table 11:** Adopted values of graphite-to-air electron mass stopping-power ratios (S¯ρ)airgraphite from Monte Carlo calculations, and differences from the previous NBS-NIST 1986 values

		^60^Co			^137^Cs			^192^Ir	
Chamber	(S¯ρ)airgraphite		Percent differences from NBS-NIST 1986	(S¯ρ)airgraphite		Percent differences from NBS-NIST 1986	(S¯ρ)airgraphite		Percent differences from NBS-NIST 1986
		values	values		values				
1cc	1.0009		+0.10	1.0096		+0.04			
10cc	1.0004		+0.10	1.0090		+0.03			
30cc	1.0002		+0.10	1.0087		+0.03			
50cc-1	1.0001		+0.10	1.0086		+0.04	1.0116		−0.33
50cc-2	1.0001		+0.10	1.0086		+0.04			
50cc-3	1.0001		+0.10	1.0086		+0.04			

**Table 12:** Factors in the humidity correction for the NBS-NIST spherical graphite ionization chambers. The results pertain to all ^60^Co, ^137^Cs, and ^192^Ir spectra considered in this report

Water vapor mass fraction %	(S¯ρ)humid airdry air	Whumid airWdry air	ρdry airρhumid air	*k*_h_
0	1.0000	1.0000	1.0000	1.0000
0.1	0.9999	0.9972	1.0008	0.9979
0.2	0.9997	0.9961	1.0014	0.9972
0.5	0.9993	0.9946	1.0033	0.9972
1.0	0.9986	0.9928	1.0062	0.9976
1.5	0.9979	0.9916	1.0092	0.9986
2.0	0.9972	0.9907	1.0123	1.0000
2.5	0.9965	0.9902	1.0153	1.0018

**Table 13:** Mass fraction percents of water vapor in humid air as a function of relative humidity (rh), temperature, and pressure. The pressures listed correspond to 740 mm Hg, 760 mm Hg (1 atmosphere), and 780 mm Hg

Temperature		20 % rh			50 % rh			80 % rh		
°C	°F	98.659 kPa	101.325 kPa	103.991 kPa	98.659 kPa	101.325 kPa	103.991 kPa	98.659 kPa	101.325 kPa	103.991 kPa
16	60.8	0.230	0.224	0.219	0.577	0.562	0.548	0.926	0.901	0.878
18	64.4	0.262	0.255	0.248	0.656	0.639	0.622	1.052	1.024	0.998
20	68.0	0.297	0.289	0.281	0.743	0.724	0.705	1.193	1.161	1.131
22	71.6	0.335	0.327	0.318	0.841	0.819	0.798	1.350	1.314	1.280
24	74.2	0.379	0.369	0.359	0.950	0.925	0.901	1.525	1.485	1.447
26	78.8	0.427	0.416	0.405	1.071	1.043	1.016	1.721	1.675	1.632

**Table 14:** Wall corrections, $k_{\text{wall}}^{\text{MC}}$, for NBS-NIST spherical graphite cavity ionization chambers from Monte Carlo calculations

Chamber	1.25 MeV photons (0 % scatter)	1.17+1.33 MeV photons (0 %)	Ehrlich et al., 5×5 cm^2^ field (14.1 %)	Ehrlich et al., 8 × 8 cm^2^ field (17.6 %)	Ehrlich et al., 10 × 10 cm^2^ field (20.9 %)	Ehrlich et al., 25 × 25 cm^2^ field (24.0 %)	Mora et al., 10 × 10 cm^2^ field (32.7 %)

^60^Co							
1cc	1.0197	1.0198	1.0202	1.0203	1.0204	1.0205	1.0207
10cc	1.0226	1.0225	1.0230	1.0231	1.0232	1.0233	1.0236
30cc	1.0249	1.0249	1.0254	1.0255	1.0257	1.0258	1.0260
50cc-1	1.0252	1.0252	1.0257	1.0258	1.0260	1.0261	1.0263
50cc-2	1.0351	1.0349	1.0355	1.0356	1.0358	1.0359	1.0363
50cc-3	1.0413	1.0413	1.0420	1.0422	1.0424	1.0425	1.0429

^137^Cs						

Chamber	0.662 MeV photons	15 % scatter spectrum	20 % scatter spectrum	25 % scatter spectrum	30 % scatter spectrum	35 % scatter spectrum
1cc	1.0286	1.0284	1.0284	1.0284	1.0285	1.0287
10cc	1.0314	1.0313	1.0313	1.0313	1.0314	1.0315
30cc	1.0348	1.0346	1.0346	1.0346	1.0347	1.0348
50cc-1	1.0349	1.0347	1.0347	1.0347	1.0348	1.0349
50cc-2	1.0468	1.0467	1.0467	1.0467	1.0468	1.0469
50cc-3	1.0533	1.0533	1.0534	1.0535	1.0537	1.0540

^192^Ir	

Chamber	^192^Ir seed at 1 m
50cc-1	1.0349

**Table 15:** Results from the calculation of deviations from cavity theory

Spectrum	(μ¯enρ)airgraphite(S¯ρ)graphiteair	α	$k_{\text{wall}}^{\left(1\right)}$	$k_{\text{wall}}^{\left(2\right)}$	*k*_cav_
^60^Co, 1.25 MeV photons	1.0023	0.0097	1.0241	1.0433	1.0000
^60^Co, 1.17 MeV + 1.33 MeV photons	1.0024	0.0096	1.0244	1.0422	1.0000
^60^Co, Ehrlich et al., 5 × 5 cm^2^ field	1.0018	0.0140	1.0249	1.0467	1.0000
^60^Co, Ehrlich et al., 8 × 8 cm^2^ field	1.0016	0.0145	1.0251	1.0469	1.0000
^60^Co, Ehrlich et al., 10 × 10 cm^2^ field	1.0015	0.0150	1.0252	1.0472	1.0000
^60^Co, Ehrlich et al., 25 × 25 cm^2^ field	1.0014	0.0154	1.0253	1.0472	1.0000
^60^Co, Mora et al., 10 × 10 cm^2^ field	1.0009	0.0183	1.0256	1.0470	1.0000

^137^Cs, 0.662 MeV photons	0.9930	0.0338	1.0374	1.0536	0.9998
^137^Cs, 15 % scatter spectrum	0.9926	0.0432	1.0371	1.0537	0.9997
^137^Cs, 20 % scatter spectrum	0.9924	0.0457	1.0370	1.0537	0.9997
^137^Cs, 25 % scatter spectrum	0.9923	0.0484	1.0369	1.0537	0.9996
^137^Cs, 30 % scatter spectrum	0.9922	0.0491	1.0370	1.0536	0.9996
^137^Cs, 35 % scatter spectrum	0.9921	0.0497	1.0372	1.0540	0.9996

^192^Ir seed, at 1 m	0.9870	0.1120	1.0358	1.0466	0.9985

**Table 16:** Adopted wall corrections $k_{\text{wall}}^{\text{MC}}$ from Monte Carlo calculations, and differences from the previous NBS-NIST 1986 values. Note that for ^192^Ir, *k*_cav_ has been incorporated in the adopted $k_{\text{wall}}^{\text{MC}}$ value

	^60^Co		^137^Cs		^192^Ir	
Chamber	$k_{\text{wall}}^{\text{MC}}$ adopted here	Percent differences from NBS-NIST1986 values	$k_{\text{wall}}^{\text{MC}}$ adopted here	Percent differences from NBS-NIST1986 values	$k_{\text{wall}}^{\text{MC}}$ adopted here	Percent differences from NBS-NIST1986 values
1cc	1.0207	+0.89	1.0285	+0.94		
10cc	1.0236	+0.70	1.0314	+0.62		
30cc	1.0260	+0.89	1.0347	+1.05		
50cc-1	1.0263	+0.85	1.0348	+0.84	1.0333	+0.18
50cc-2	1.0363	+0.94	1.0468	+0.91		
50cc-3	1.0429	+0.91	1.0537	+0.77		

**Table 17:** NIST gamma-ray beam sources

Radionuclide	Nominal activity (1 Jan 03) (Bq)	Location^a^ (room)	Beam orientation
^60^Co	3.3 × 10^14^	B034	vertical
^60^Co	9.6 × 10^13^	B036^b^	vertical
^60^Co	7.7 × 10^10^	B021B	horizontal
^60^Co	5.7 × 10^9^	B015B	horizontal
^137^Cs	2.8 × 10^13^	B036	vertical
^137^Cs	5.1 × 10^12^	B021A	horizontal
^137^Cs	5.8 × 10^11^	B015A	horizontal

a Room in NIST’s Radiation Physics building (Bldg. 245).

b Source replaced November 1999; previous source activity would be ≈1.8 × 10^13^ Bq.

**Table 18:** Changes in the NIST exposure and air-kerma standards based on the results of calculations reported here

			Using the changes due only to the new wall corrections, $\bar{R}=1.0088$			Including also the changes in $\bar{g}$, the photon mass energy-absorption and electron mass stopping-power ratios, and the humidity correction, $\bar{R}=1.0105$		
Chamber					Relative change from			Relative change from
	*ω_j_*	*_j_k*_std_	*R_j_*	${k^{\prime }}_{j\;\text{std}}$	${k^{\prime }}_{j\;\text{std}}$	*R_j_*	${k^{\prime }}_{j\;\text{std}}$	${k^{\prime }}_{j\;\text{std}}$

^60^Co								
1	0.02928	0.9970	1.0089	0.9969	–0.01 %	1.0106	0.9969	–0.02 %
10	0.03304	0.9997	1.0070	1.0015	+0.18 %	1.0087	1.0015	+0.17 %
30	0.23347	1.0005	1.0089	1.0004	–0.01 %	1.0106	1.0004	–0.01 %
50-1	0.26527	1.0002	1.0085	1.0005	+0.03 %	1.0102	1.0005	+0.03 %
50-2	0.22757	1.0003	1.0094	0.9997	–0.06 %	1.0111	0.9997	–0.07 %
50-3	0.21137	0.9998	1.0091	0.9995	–0.03 %	1.0108	0.9995	–0.04 %

^137^Cs								

			Using the changes due only to the new wall corrections, change from $\bar{R}=1.0088$			Including also the changes in $\bar{g}$, the photon mass energy-absorption and electron mass stopping-power ratios, and the humidity correction, $\bar{R}=1.0090$		
Chamber					Relative change from			Relative change from
	*ω_j_*	*_j_k*_std_	*R_j_*	${k^{\prime }}_{j\;\text{std}}$	${k^{\prime }}_{j\;\text{std}}$	*R_j_*	${k^{\prime }}_{j\;\text{std}}$	${k^{\prime }}_{j\;\text{std}}$
1	0.02928	0.9970	1.0094	0.9964	–0.06 %	1.0097	0.9963	–0.07 %
10	0.03304	0.9997	1.0062	1.0022	+0.25 %	1.0064	1.0023	+0.26 %
30	0.23347	1.0005	1.0105	0.9988	–0.17 %	1.0107	0.9988	–0.17 %
50-1	0.26527	1.0002	1.0084	1.0006	+0.04 %	1.0087	1.0005	+0.03 %
50-2	0.22757	1.0003	1.0091	1.0000	–0.03 %	1.0094	0.9999	–0.04 %
50-3	0.21137	0.9998	1.0077	1.0009	+0.11 %	1.0080	1.0008	+0.10 %

^192^Ir				

	Using the changes due only to the new wall corrections,		Including also the changes in $\bar{g}$, the photon mass energy-absorption and electron mass stopping-power ratios, and the humidity correction	
Chamber	*R*	Relative change	*R*	Relative change
50-1	1.0018	+0.18 %	0.9994	–0.06 %

## Footnotes

1 Evaluated in the continuous-slowing-down approximation [15].

2 A sphere is an isotropic detector for any angular distribution uniform over its surface. A point-isotropic source at some distance, however, has angles of incidence correlated with the entry-point location on the chamber. For a source-detector distance of ≳100 cm corresponding to a typical ^60^ Co or ^137^Cs calibration, one might expect the beam to be nearly parallel. Rogers and Treurniet [8], using Monte Carlo calculations, report axial non-uniformity corrections for NIST chambers of less than 0.05 %, except for the 1cc chamber in a ^137^Cs beam for which they indicate a correction of −0.12 ± 0.06 %. This small correction will be neglected for our purposes.

3 First collisions in the cavity air were included in the scores, even though such events are not part of simple cavity theory. From simple considerations of the mean chord lengths in both the graphite shell and air cavity, it is estimated that for the higher-energy incident photons the number of primary photon interactions in the air cavity is less than 0.1 % of those in the graphite shell. This rather small contribution is not expected to significantly affect the results for ^60^Co and ^137^Cs beams. Further attention to this issue will be given later, mainly for ^192^Ir gamma rays.

4 Certain commercial equipment, instruments, or materials are identified in this paper to foster understanding. Such identification does not imply recommmendation or endorsement by the National Institute of Standards and Technology, nor does it imply that the materials or equipment identified are necessarily the best available for the purpose.

5 This intermediate value is a weighted mean obtained by including the Bichsel-Hiraoka value with the previous pertinent determinations of the mean excitation energy for graphite.

6 Using the fluence spectra in a thin graphite-shell region at the graphite-air interface would perhaps be more consistent with cavity theory.

7 The equation appears to be in essential agreement with the work of Jones [33, 34].

8 There appears to be some confusion in Table 1 of Rogers and Treurniet [8]: the graphite wall thicknesses and outer radii are listed correctly, but they give inner radii that are the *sum* of the outer radii and the wall thicknesses instead of the *difference*. It is assumed that this is simply a typographic error.

9 Their value includes a small axial non-uniformity correction for the NIST chambers of 1.0001.
